# The Space-Exposed Kombucha Microbial Community Member *Komagataeibacter oboediens* Showed Only Minor Changes in Its Genome After Reactivation on Earth

**DOI:** 10.3389/fmicb.2022.782175

**Published:** 2022-03-11

**Authors:** Daniel Santana de Carvalho, Ana Paula Trovatti Uetanabaro, Rodrigo Bentes Kato, Flávia Figueira Aburjaile, Arun Kumar Jaiswal, Rodrigo Profeta, Rodrigo Dias De Oliveira Carvalho, Sandeep Tiwar, Anne Cybelle Pinto Gomide, Eduardo Almeida Costa, Olga Kukharenko, Iryna Orlovska, Olga Podolich, Oleg Reva, Pablo Ivan P. Ramos, Vasco Ariston De Carvalho Azevedo, Bertram Brenig, Bruno Silva Andrade, Jean-Pierre P. de Vera, Natalia O. Kozyrovska, Debmalya Barh, Aristóteles Góes-Neto

**Affiliations:** ^1^Laboratory of Molecular and Computational Biology of Fungi, Department of Microbiology, Department of Genetics, Ecology and Evolution, Institute of Biological Sciences, Federal University of Minas Gerais, Belo Horizonte, Brazil; ^2^Laboratory of Cellular and Molecular Genetics, Department of Genetics, Ecology and Evolution, Universidade Federal de Minas Gerais, Belo Horizonte, Brazil; ^3^Postgraduate Program in Biology and Biotechnology of Microorganisms, Department of Biological Sciences, State University of Santa Cruz, Ilhéus, Brazil; ^4^Computational Biology and Biotechnological Information Management Center (NBCGIB), State University of Santa Cruz, Ilhéus, Brazil; ^5^Institute of Molecular Biology and Genetics of NASU, Kyiv, Ukraine; ^6^Department of Biochemistry, Genetics and Microbiology, Centre for Bioinformatics and Computational Biology, University of Pretoria, Pretoria, South Africa; ^7^Center for Data and Knowledge Integration for Health (CIDACS), Institute Gonçalo Moniz, Oswaldo Cruz Foundation (FIOCRUZ-Bahia), Salvador, Brazil; ^8^Institute of Veterinary Medicine, Burckhardtweg, University of Göttingen, Göttingen, Germany; ^9^Laboratory of Bioinformatics and Computational Chemistry, Department of Biological Sciences, State University of Southwest Bahia (UESB), Jequié, Brazil; ^10^German Aerospace Center (DLR) Berlin, Institute of Planetary Research, Planetary Laboratories, Astrobiological Laboratories, Berlin, Germany; ^11^Centre for Genomics and Applied Gene Technology, Institute of Integrative Omics and Applied Biotechnology, Purba Medinipur, India

**Keywords:** comparative genomics, Acetobacteraceae, metabolic reconstruction, single nucleotide variation, nitrogen fixation, hopanoids, cellulose biosynthesis

## Abstract

*Komagataeibacter* is the dominant taxon and cellulose-producing bacteria in the Kombucha Microbial Community (KMC). This is the first study to isolate the *K. oboediens* genome from a reactivated space-exposed KMC sample and comprehensively characterize it. The space-exposed genome was compared with the Earth-based reference genome to understand the genome stability of *K. oboediens* under extraterrestrial conditions during a long time. Our results suggest that the genomes of *K. oboediens* IMBG180 (ground sample) and *K. oboediens* IMBG185 (space-exposed) are remarkably similar in topology, genomic islands, transposases, prion-like proteins, and number of plasmids and CRISPR-Cas cassettes. Nonetheless, there was a difference in the length of plasmids and the location of *cas* genes. A small difference was observed in the number of protein coding genes. Despite these differences, they do not affect any genetic metabolic profile of the cellulose synthesis, nitrogen-fixation, hopanoid lipids biosynthesis, and stress-related pathways. Minor changes are only observed in central carbohydrate and energy metabolism pathways gene numbers or sequence completeness. Altogether, these findings suggest that *K. oboediens* maintains its genome stability and functionality in KMC exposed to the space environment most probably due to the protective role of the KMC biofilm. Furthermore, due to its unaffected metabolic pathways, this bacterial species may also retain some promising potential for space applications.

## Importance

Nowadays, there is an increased effort to study the possibility of life on Mars and the ability of organisms to survive the Mars environment to assess the possibility of colonizing the planet. Recent studies have shown that bacterial cellulose is a strong material that has a variety of uses and applications, such as medicine, tissue regeneration, fabrics, electronics, and biotechnology in general. In this study we were able to expose a cellulose producing bacteria (*Komagataeibacter oboediens*) to the space/Mars-like environment, and our results show that *K. oboediens* was able to survive being exposed to the space environment for 18 months and that its genome remains mainly conserved after spaceflight. This suggests that *K. oboediens* is a strong candidate for cellulose production in Mars colonies’ industry.

## Introduction

One of the most intriguing questions is the capability of an organism to survive and resist the extremely harsh extraterrestrial outer space conditions, such as cosmic radiation, microgravity, temperature extremes, substrates, atmospheric pressure, and vacuum (Horneck et al., 2010; de Vera et al., 2014; Schulze-Makuch et al., 2015). In order to find an answer, the European Space Agency (ESA) conducted a series of multi-user space biology exposure experiments, using the EXPOSE-R2 platform installed outside the International Space Station (ISS) that provides an environment for sets of long-term experiments (1.5 years). One of the biological samples elected to be sent to the ISS was dehydrated kombucha biofilm in the project BIOMEX (BIOlogy and Mars EXperiment) (de Vera et al., 2019; de Vera, 2020).

Kombucha multimicrobial community (KMC) is a natural assemblage of bacteria and yeasts living in metabolic cooperation and streaky in a cellulose-based pellicle film (Pothakos et al., 2016; May et al., 2019; Barbosa et al., 2021). In astrobiology, KMC is used as a model for researching a natural probiotic drink for astronauts during space travel (Kozyrovska et al., 2012, 2021). Recent BIOMEX results revealed that key KMC community members were capable of surviving under space and Mars-like conditions (Podolich et al., 2019) and that the bacterial cellulose (BC) produced by KMC retained its robustness under the impact of Mars-like stressors (Orlovska et al., 2021). Our post-flight metagenomic studies proved that the genus *Komagataeibacter* was the dominant bacterial taxon both in the ground-based reference KMC ecotype and reactivated cultured KMC samples exposed to Mars-like conditions outside the ISS (Góes-Neto et al., 2021; Lee et al., 2021). Moreover, we observed that the key KMC strain *K. oboediens*, which survives under such extraterrestrial stresses, retains its cellulose synthesizing *bcs* operon genes intact (Góes-Neto et al., 2021). This observation led us to analyze the entire *K. oboediens* genome from space-exposed KMC samples to understand its genome stability under extra-terrestrial conditions. Little is known about the *K. oboediens* genome, and for this reason, herein, we isolated, sequenced, and characterized the *K. oboediens* IMBG180 (from ground based KMC culture) and *K. oboediens* IMBG185 (from space exposed KMC samples) and compared their genomes to understand their genomic components. In this study, we addressed the question if there are significant differences between *K. oboediens* strains under post-flight space/Mars-like and standard terrestrial conditions by: (i) identifying and analyzing all the shared and exclusive genes, (ii) evaluating Single Nucleotide Variations (SNVs) in protein-coding genes, and (iii) performing global metabolic reconstruction of the two strains.

## Materials and Methods

### Exposure Conditions, Reactivation of Space-Returned Kombucha Microbial Community Samples, and Bacterial Strains

**FIGURE 1:**
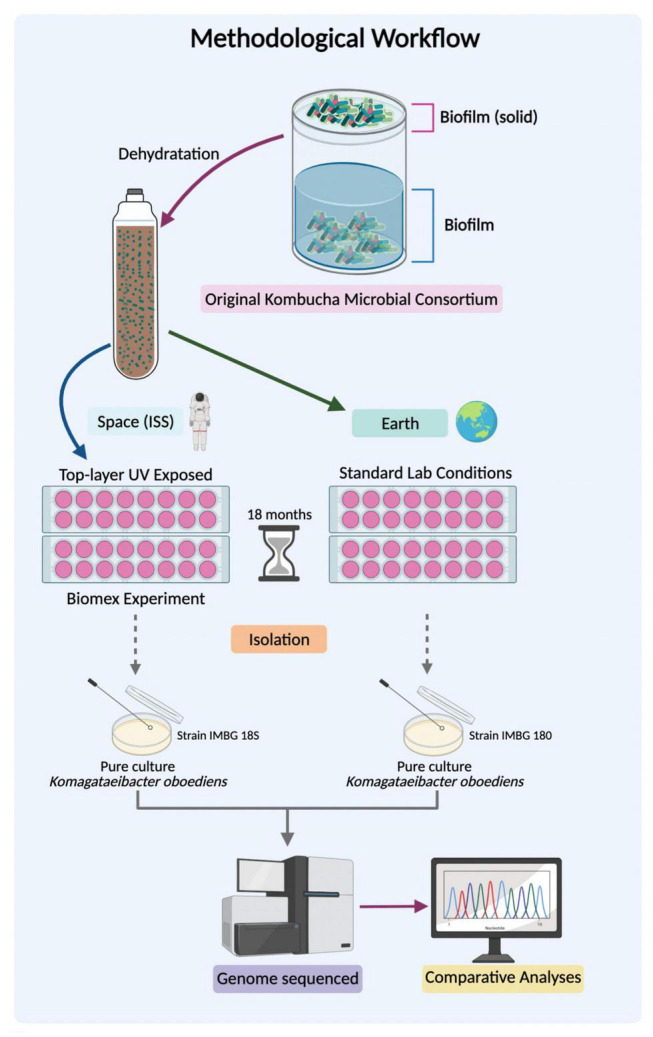
Methodological workflow.

All the methodological workflow (from the original KMC consortium until the genome sequencing of isolated strains) is graphically depicted as Figure 1. The original kombucha microbial culture was obtained from the collection of microorganisms of the Institute of Molecular Biology and Genetics (Kyiv, Ukraine). It was maintained in filter-sterilized black tea (Lipton, 1.2%, w/v) with white sugar (3%, w/v) (BTS) at 28°C, prior to preparation as dry organo-mineral KMC pellicle fragments samples previously described in Podolich et al. (2019). The KMC samples were placed in a three-level tray hosting four samples (independent sampling units or biological replicates) on either level (top, middle, and bottom) and mounted on an EXPOSE-2 platform outside the ISS as described in de Vera et al. (2019). These samples were maintained for 18 months (1.5 years) within the tray under a simulated Mars atmosphere (95.55% CO_2_, 2.70% N_2_, 1.60% Ar, 0.15% O_2_, ∼370 ppm H_2_O), under pressure of 980 Pa. No UV filters were used in the kombucha experiments, and space varied between 4.5 × 10^6^ and 8.4 × 10^6^ kJ/m^2^ (de Vera et al., 2019). A set of analogous samples were kept in the laboratory at room temperature in darkness. Returned and ground reference KMC samples were cultivated for reactivation in filter-sterilized sugared (7%) black tea infusions at 28°C until a cellulose-based pellicle appeared, meaning that bacteria from the genus *Komagataeibacter* were active. In reference samples, pellicles appeared in 7 days, and returned samples formed pellicles after 60 days of a culturing. After KMC sample reactivation, *K. oboediens* IMBG185 was isolated from the exposed top-level KMC samples and opened to UV irradiation, and *K. oboediens* IMBG180 from initial reference KMC (iKMC) was kept in the lab during the spaceflight experiment, desiccated and stored at room temperature. Both samples were sequenced together to avoid variation due to different sequencing runs. Both strains were cultured in the HS medium (Hestrin and Schramm, 1954) at 28°C for 3 days before extraction of genomic DNA.

### Genomic DNA Extraction, Library Construction, and Whole-Genome Sequencing

Total DNA from the planktonic cells of KMC was isolated by using the innuSPEED Bacteria/Fungi DNA isolation kit (Analytik Jena AG, Germany) during the stage of biofilm formation (7th day). The nucleic acids were quantified and qualified by a NanoDrop ND-1000 spectrophotometer (NanoDrop Technologies, Wilmington, DE, United States). A 450 bp library was prepared from genomic DNA with the NEBNext Fast DNA Fragmentation and Library Preparation Kit (New England Biolabs, Ipswich, MA, United States) following the manufacturer’s instructions. A library of 450 bp was prepared with the NEBNext Fast DNA Fragmentation and Library Preparation Kit (New England Biolabs). Library quality was checked with Agilent 2100 Bioanalyzer. The genomes were sequenced using Illumina HiSeq 2500 (Illumina, San Diego, CA, United States) to obtain paired-end reads. The obtained final genome coverage of IMBG185 (715.0x) and IMBG180 (1964.0x).

### Genome Assembly

**FIGURE 2:**
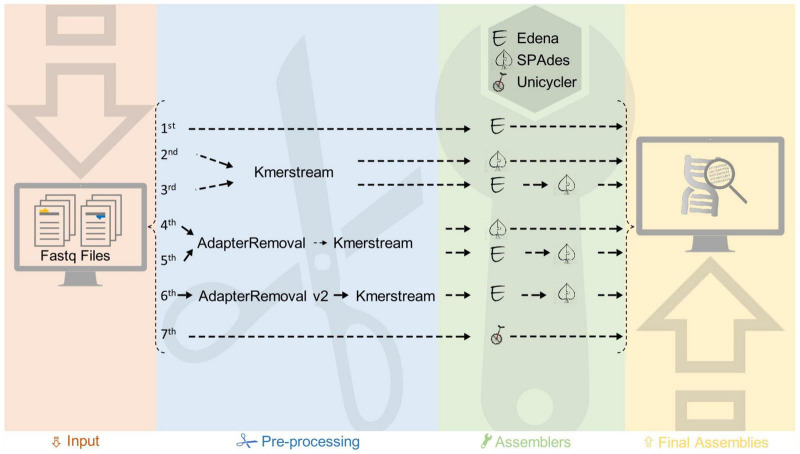
Steps of the customized pipeline used for assembly of ground reference (IMBG180) and space/Mars-like conditions (IMBG185) genomes.

*De novo* genome assemblies of *K. oboediens* strains were carried out with a customized pipeline (Figure 2). Using this pipeline, the read quality was checked using FastQC v0.11.8^1^, followed by seven different assembly strategies, executed with distinct software and parameters. The first assembly was carried out by Edena v3.131028 (Hernandez et al., 2008), in which the overlap-layout-consensus approach was employed to assemble the raw paired short reads solely exploiting the overlaps between them. The second assembly was executed using SPAdes v3.13 (Bankevich et al., 2012), which breaks the raw paired short reads into k-mers (substrings of size k) and applies De Bruijn graphs, which were generated to perform genome assemblies. The k-mers used for this second assembly were the best three predicted by KmerStream version 1.1 (Melsted and Halldórsson, 2014). The third assembly is a hybrid between Edena and SPAdes, where the contigs produced in the first assembly were employed as trusted contigs in a new assembly performed by SPAdes, again using the raw paired short reads and the three best k-mers detected by KmerStream. In the fourth assembly, SPAdes was executed again after quality control of the short reads performed by AdapterRemoval v2 (Schubert et al., 2016) and k-mer prediction using KmerStream. AdapterRemoval was employed to collapse paired short reads that overlap by at least 11 base pairs, remove adapters, and trim base pairs from the 3′ end with quality lower than Phred 20 and, ultimately, exclude short reads that were left with less than 31 nucleotides after trimming. Trimmed paired reads were passed as parameters to SPAdes in conjunction with the singleton and collapsed short reads. The singleton and collapsed short reads were also used in the fifth assembly, but this time, the raw paired reads were used as parameters to SPAdes besides the contigs produced by Edena, which were used as trusted contigs. The k-mers used were the top 3 predicted by KmerStream. In the sixth assembly, which was also performed by SPAdes, we employed the singleton, collapsed, and paired short reads enhanced by AdapterRemoval along with the contigs assembled by Edena, which were used as trusted contigs. The top 3 k-mers predicted by KmerStream were also used for the last assembly. The seventh assembly was performed by Unicycler v.0.4.5 and Spades v.3.9.1. Afterward, we ran QUAST (Gurevich et al., 2013) to evaluate these seven distinct assemblies for each genome, considering the N50 score, lesser numbers of contigs, and the expected genome size. Finally, the genome obtained from Unicycler v. 0.4.5 was considered as the best assembled for each of the two genomes (IMBG180 and IMBG185).

### Genome Annotation

The genome sequences were annotated using Prokka v. 1.14.5 prokaryotic genome annotation (Seemann, 2014), as well as using BUSCO v. 4.0.1 to predict the completeness of single-copy orthologs (Seppey et al., 2019), and MAUVE v. 2.4.0 (Darling et al., 2004) to verify the synteny of the genes between the two genomes. The predicted contigs were also analyzed with GO FEAT (Araujo et al., 2018), an on-line platform for functional annotation and enrichment of genomic data integrated with UNIPROT^2^, INTERPRO^3^, PFAM^4^, NCBI^5^, and KEGG^6^ databases.

The draft genomes of *K. oboediens* strains, both the space/Mars-like conditions (IMBG185) and the reference ground (IMBG180), were deposited in NCBI with the following accession numbers: *K. oboediens* IMBG185 (BioProject: PRJNA632016, BioSample: SAMN14942470) and *K. oboediens* IMBG180 (BioProject: PRJNA633374, BioSample: SAMN14942824, Accession: JABLUV000000000), respectively.

### Taxonomic Identification, Phylogenetic (Small-Scale), and Phylogenomic (Large-Scale) Analyses

For species identification, a multilocus sequence analysis (MLSA), a small-scale phylogenetic analysis, was performed based on concatenated partial sequences of the housekeeping genes: 16S rRNA, *dnaK*, *rpoB*, and *groEL*, as previously described (Liu et al., 2018). These gene sequences were extracted from the strains IMBG185 and IMBG180 of deposited genomes and other 16 *Komagataeibacter* species (Table 1) from NCBI and aligned with MAFFT v7.310 (Katoh and Standley, 2013). *Acetobacter aceti* was used as the outgroup. The phylogeny was reconstructed using the maximum likelihood method implemented in IQ-TREE v1.6.12 (Nguyen et al., 2015), and the robustness of the branches was evaluated by bootstrap analysis based on 1000 iterations. For large-scale phylogenomic analysis, MUMmer (NUCmer) was used to align the genome sequences, and Pyani tool version 0.2.x was used to calculate the percentage nucleotide identity for the analysis of average nucleotide identity (ANI) and to perform the biplot dendrogram associated to a color matrix.

**TABLE 1:** *Komagataeibacter* spp. gene sequences from GenBank (NCBI) used for phylogeny reconstruction of ground reference (IMBG180) and space/Mars-like conditions (IMBG185) genomes.

Species*	16S	*dnaK*	*groEL*	*rpoB*
*K. cocois*	KR998072	KR998073	KR998074	KR998075
*K. diospyri*	MG971333	MK306725	MK306727	MK30679
*K. europaeus*	JF793984	FN391614	FN391687	FN391760
*K. hanseni*	JF793987	FN391617	FN391690	FN391763
*K. intermedius*	JF793990	FN391620	FN391693	FN391766
*K. kakiaceti*	AB607833	JX022617	JX022618	JX022619
*K. kombuchae*	AY688433	FN391624	FN391697	FN391770
*K. maltaceti*	AB680671	HE866297	HE866298	HE866299
*K. medellinensis*	AB680048	JX013854	JX013856	JX013858
*K. nataicola*	JF794000	FN391628	FN391701	FN391774
*K. oboediens*	JF794004	FN391631	FN391704	KC478395
*K. rhaeticus*	JF794006	FN391633	FN391706	FN391779
*K. saccharivorans*	JF794010	FN391635	FN391708	FN391781
*K. sucrofermentans*	AB598742	FN391639	FN391712	FN391785
*K. swingsii*	JF794011	FN391638	FN391711	FN391784
*K. xylinus*	JF794013	FN391641	FN391714	FN391787
Acetobacter aceti**	NR026121	KF537399	KC176424	KF537487

*Some Komagataeibacter spp. are originally annotated as the former Glucanoacetobacter genus.

**Used as outgroup.

### Prediction of Genomic Islands, Transposases, and Prion Aggregating Regions

The prediction of genomic islands and transposases in the genomes were performed using the Genomic Islands Prediction Software GIPSy (Soares et al., 2016), using *K. saccharivorans* strain CV1 as a reference genome. This tool provides information about the genes present in these islands (symbiosis, pathogenicity and virulence, antibiotic resistance, and metabolic) and their respective location in the genomes. Only genomic islands with normal and strong classifications were considered. In addition, GIPSy also detects transposase genes based on HMMER3 (Eddy, 2011) with *E*-value ≤ 1 × 10^–4^. The online tool PAPA (Prion aggregation prediction algorithm) (Toombs et al., 2012) was used to predict candidate prion aggregating regions in the sequences annotated as “Transcription termination factor Rho” in both genomes.

### Prophage and Plasmid Characterization

Prophage Hunter tool (Song et al., 2019) was used for prediction and classification of prophages. Circular contigs with high depth coverage, predicted by Unicycler (Wick et al., 2017), were considered plasmids. Non-circular contigs were assessed using a combination of three different tools: (i) Mob-recon (reference-based approach, version 2.1.0) (Robertson and Nash, 2018), (ii) HyAsP (hybrid approach, version 1.0.0) (Müller and Chauve, 2019), and (iii) Gplas (version 0.5.0) (Arredondo-Alonso et al., 2020). Non-circular contigs were considered plasmids only when predicted by the majority of these three tools.

### Prediction of CRISPR-Cas

The prediction of Cas proteins and classification of CRISPR-Cas system subtype in both genomes were performed via CRISPRCas Identifier (Padilha et al., 2020).

### Evaluation and Integration of the Metabolic Repertoire of *Komagataeibacter oboediens*

The KAAS web server (Moriya et al., 2007) was used to predict KEGG Orthology (KO) identifiers for both the sequenced strains. The representative set of organisms was selected as the default for prokaryotes, in addition to the closely related species *Gluconacetobacter diazotrophicus* (three-letter code: *gdi*), *Acetobacter pasteurianus* (*apt*), and *Granulibacter bethesdensis* (*gbe*). The bidirectional best-hit approach was used as an assignment method. In order to complement KEGG analysis, the MetaCyc (Caspi et al., 2020) ontology was also employed to perform metabolic prediction. For this analysis, the replicons and associated annotation for both genomes were input to Pathway Tools (Karp et al., 2002), which creates a Pathway/Genome Database (PGDB) containing the predicted metabolic pathways for the organisms. Metabolic reconstruction included determining gene-protein-reaction associations, which are primarily based on the corresponding enzyme commission (EC) numbers. The pathway score yielded by Pathway Tools was used as a proxy for the presence or absence of a given pathway.

Pathway Tools was also employed to thoroughly study the key cellulose synthesis *bcs* operon, which was mined in *K. oboediens* using the *bcs* operon identified in *K. xylinus* as template (PMID: 31983038; PMID: 30761107). BLAST analyses against the UniProt database (PMID: 15215342) were also performed to search for orthologs related to this metabolic function.

Stress-related genes and pathways were identified by manual literature searches as well as by the retrieval of curated proteins deposited in UniProtKB/Swiss-Prot, which were associated to the “Stress response” keyword (KW-0346) and belonged to either Archaea or Bacteria domains. In order to reduce the redundancy of this protein set (originally composed of 7,724 sequences), the proteins were clustered at 90% sequence identity using UniRef90 clusters, yielding a final set of 2,687 stress-related sequences. Subsequently, for retrieving orthologs in *K. oboediens*, BLASTp was performed using each predicted *K. oboediens* proteome set as a query against the reference stress-related proteins, followed by manual curation of the alignments.

A principal component analysis (PCA) was also conducted to assess the metabolic profiles of *K. oboediens* and related bacteria. In order to perform this task, the enzymatic content (as predicted by KEGG) was used as input to the *prcomp* function in R^7^, which employs the singular value decomposition method to extract principal components. The compared bacteria (and corresponding labels, when applicable) were *Acetobacter aceti* (Aac), *A. cerevisae* (Ace), *A. malorum* (Ama), *A. pasteurianus* (Apa), *A. pomorum* (Apo), *Clostridium kluyveri, Escherichia coli, E. albertii, E. fergusonii, Komagataeibacter cocoi* (Kco), *K. diospyri* (Kdi), *K. europaeus* (Keu), *K. hansenii* (Kha), *K. intermedius* (Kin), *K. kakiaceti* (Kka), *K. maltaceti* (Kma), *K. medellinensis* (Kme), *K. nataicola* (Kna), *K. oboediens* LMG 18849 (Kob 18849), *K. rhaeticus* (Krh), *K. saccharivorans* (Ksa), *K. sucrofermentais* (Ksu), *K. swingsii* (Ksw), *K. kylinus* (Kxy), *Salmonella bongori, S. enterica, Shigella boydii, Sh. flexneri*, and *Sh. sonnei*.

### Single Nucleotide Variation Analysis

A comparison between coding sequences (CDS), as well as corresponding plasmid CDS, of *K. oboediens* in space and terrestrial conditions was performed to search for SNV between their genomes and their plasmids. An in-house Python script (see Availability of data and materials) was used to find the corresponding genes in both genomes based on the following criteria: (i) both genes had to present exactly the same 60 first nucleotides and (ii) have the same gene length. These criteria were applied assuming that the space stress would not have caused severe mutations that would make these criteria to fail the analysis, as such stress would possibly be lethal to the bacteria.

Furthermore, a list of corresponding pairs of genes present in both genomes was generated, as well as a list of genes without a match in the other genome. The list of genome-specific, putative exclusive genes was then further manually curated. The lists generated are available in Supplementary Material 1.

### Protein Modeling and Molecular Dynamics

In order to evaluate the *K. oboediens* pyruvate phosphate dikinase (ppdk) single mutation amino acid, we performed ppdk homology modeling and molecular dynamics for discovering the influence of the amino acid SER287 on the ATP/ADP site. Firstly, the *K. oboediens* PPDK amino acid FASTA sequence was submitted to The Swiss Model Workspace (Arnold et al., 2006) for automated modeling using the PDB template 5LU4^8^, because it is complexed with the ADP molecule, as well as Mg^++^ cofactor. Additionally, we have considered the template resolution below 3.0 Å, identity, and sequence coverage above 50%. After QMEAN validation, we submitted both the ppdk model and its template to a structural alignment using the UCSF Chimera (Pettersen et al., 2004) program to identify the catalytic and other important binding site positions, as well as to verify if the ppdk mutation could affect the enzyme function. Considering the ATP/ADP binding site’s high identity between both the ppdk model and its 5LU4 template, and, since ppdk SER287 was aligned with 5LU4 GLY285, we decided to generate a single mutation in 5LU4 (GLY285 to SER285) in order to use this crystallographic structure complexed with ADP and Mg^++^ ligands and performed a molecular dynamics simulation for describing how this mutation would affect the ATP/ADP binding site, which would be important for enzyme activation and its transition state (Minges et al., 2017b). In this case, using a homologous crystallographic structure avoids a possible bias, which could happen only if a modeled protein docked with its respective ATP/ADP ligand was used.

For the Molecular Dynamic experiments, we used the GROMACS 2020 program. The mutated complex 5LU4, bound to ADP and Mg^++^, was subjected to a stability MD in water solvent for 50 ns. Subsequently, we followed the GROMACS tutorial for protein–ligand complexes (Lemkul, 2019) with adaptations, as well as with the program manual (Berendsen et al., 1995) for all the simulation steps and analyses. The MD graphs were generated using the Grace 5.1.25 program^9^, and the output data were used for creating a video of the simulation using the VMD program (Humphrey et al., 1996).

## Results

### IMBG180 and IMBG185 Belong to *Komagataeibacter oboediens*

**FIGURE 3:**
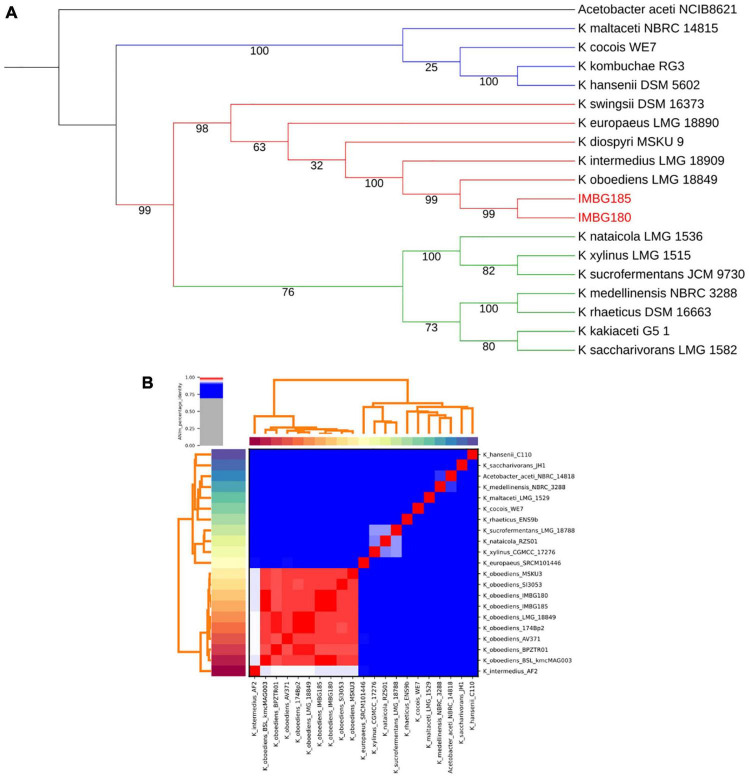
**(A)** Maximum likelihood phylogenetic tree of IMBG185 and IMBG180 strains and *Komagataeibacter* species from GenBank (NCBI). The tree represents concatenated partial sequences of the housekeeping genes, 16S *rRNA*, *dnaK*, *rpoB*, and *groEL*. Bootstrap support values (1,000 replicates) are indicated below the branches. Clade 1 (blue); Clade 2 (red + green); Clade 3 (green); Clade 4 (red). **(B)** Large-scale (phylogenomics) global similarity analysis (ANI) of IMBG185 and IMBG180 strains and available *Komagataeibacter* species from GenBank (NCBI). The large red cluster contains all the genomes of the most globally similar *Komagataeibacter* species, which includes IMBG185 and IMBG180 strains and all the available *Komagataeibacter oboediens*.

A total of two distinct and highly supported clades (bootstrap values: 99–100%) were retrieved in multigene phylogenetic analysis of the genus *Komagataeibacter*: one of them (clade 1), including the well-studied and representative species, *K. hansenii*, and the other one (clade 2) comprising another much studied and representative species, *K. xylinus*. Clade 2, in turn, encompassed two differently and highly supported clades: clade 3, with 76% bootstrap, including, besides *K. xylinus* and other related species, *K. medellinensis* and *K. saccharivorans*, and clade 4, encompassing *K. oboediens*, *K. intermedius* and related species (Figure 3A). Moreover, the analysis clearly revealed that both strains (IMBG180 and IMBG185) are very closely related to *K. oboediens* (Figure 3A), thus confirming that they belong to the same species *K. oboediens*. Furthermore, this finding of the small-scale phylogenetic analysis (MLSA) was fully corroborated with large-scale phylogenomic analysis based on global genomic similarity (ANI), showing that both strains (IMBG180 and IMBG185) and all the *K. oboediens* available genomes are the most identical (the large red cluster) amongst all the other genomes of *Komagataeibacter* species (Figure 3B). Moreover, ANI (average nucleotide identity) index between *K. oboediens* IMBG180 and IMBG185 were 99.9%, suggesting that they are almost identical.

### Notable Similarities Between *Komagataeibacter oboediens* IMBG180 and IMBG185 Genomes

**FIGURE 4:**
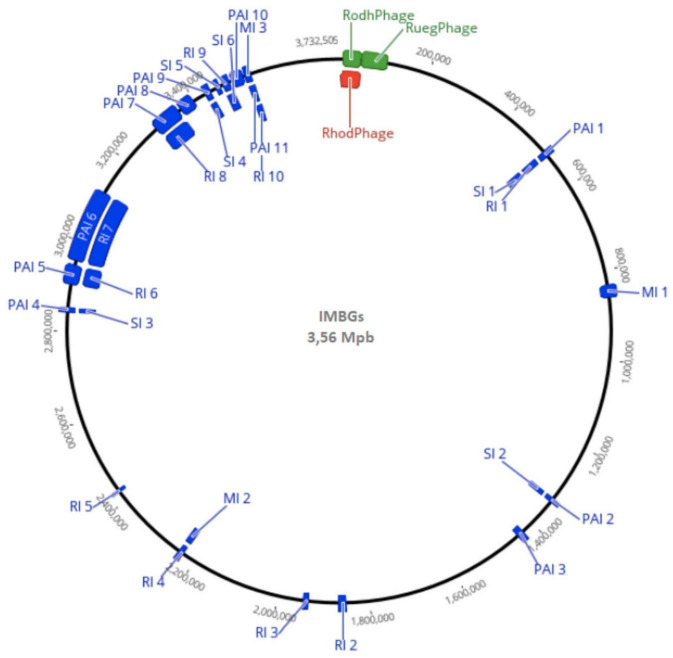
General whole genome representation of IMBGs (IMBG180 – ground reference, and IMBG185 – space/Mars-like conditions), with Genomic Islands highlighted in blue (MI: Metabolic, PI: Pathogenic Islands, RI: Resistance Islands, and SI: Symbiotic Islands) and active prophages highlighted in green (IMBG180) and red (IMBG185). RhodPhage: *Rhodovulum* phage RS1; RuegPhage: *Ruegeria* phage DSS3-P1.

A remarkable similarity between IMBG185 (space/Mars-like conditions) and IMBG180 (ground reference) strains of sequenced genomes (Figure 4 and Supplementary Material 2) was noticed. Very small differences in size (3,560,605 and 3,562,447 bp), total gene prediction (3,282 and 3,275), total number of protein-coding genes (3,230 and 3,222), and other features between samples IMBG185 and IMBG180, respectively, may be because they are not of the same strain or because of minor changes in IMBG185 due to the stressful conditions it was exposed to. Besides, both strains displayed exactly the same G + C content (61.85%) and the same number of rRNA genes (3) (Table 2).

**TABLE 2:** General features of *K. oboediens* strains of ground reference (IMBG180) and space/Mars-like conditions (IMBG185) genomes.

Genomic features	*K. oboediens* strains	
	IMBG180	IMBG185
Size of the chromosome (bp)	3,562,447	3,560,605
Contigs	62	59
G + C content (%)	61.85	61.85
Total genes	3,275	3,282
Protein coding genes	3,222	3,230
rRNA genes	3	3
tRNA genes	49	48
tmRNA	1	1

### Both Genomes Have 427 Orthologous Genes

In the genome completeness analysis, 432 orthologous genes were found in the two analyzed genomes (IMBG185 and IMBG180), according to the following annotation databases: tBLASTn 2.2+, Augustus 3.2, Prodigal, and HMMER3.1. The genomes also showed 427 complete orthologous genes divided by categories: singles-copy (425), duplicated (2), fragmented (1), and missing (1). These results revealed the very high similarity between IMBG185 and IMBG180 sequenced genomes (Supplementary Material 2).

### Identical Genomic Islands, Transposases, and Prion-Like Proteins

The number of symbiosis (4), metabolic (4), and virulence + pathogenicity islands (7), and antibiotic resistance (6) islands found in both IMBGs were identical. Furthermore, considering only the identifications with identity ≥ 30% and *E*-values ≤ 1 × 10^–7^, the number of protein-coding genes inside all the genomic islands were also similar (Figure 4 and Supplementary Material 3). Moreover, the prediction of transposases retrieved 39 potential protein sequences for both IMBGs. For Prion-like proteins, the transcription terminator Rho gene had been detected in both IMBGs, but the Cb-Rho cPrD (prion-forming) domain was not found in the IMBG180 and IMBG185 genomes. This may mean that *Cb*-Rho does not behave as a prion in IMBGs.

### IMBG180 and IMBG185 Genomes Show Very Little Difference in Number of Prophages

A total of 96 candidates for prophage regions in the IMBGs were predicted, and these regions have 92 inactive, three active, and one ambiguous prophage (Figure 3 and Supplementary Material 4). Regarding the regions containing active prophages, 62 genes found in IMBGs displayed high similarity to *Rhodovulum* phage RS1 (score = 0.87), and 52 genes from postflight (IMBG185) and 60 genes from reference (IMBG180) genomes showed high similarity to *Ruegeria* phage DSS3-P1 (score = 0.97). Moreover, 18 genes from the genome IMBG185 contained similar sequences to *Streptomyces* phage (score = 0.92), whereas in the IMBG180 genome, 19 prophage genes in unrelated regions with other bacterial species were also characterized.

### Both Genomes Have Two Plasmids but Differ in Sequence Length

Plasmid prediction of the assembled contigs of *K. oboediens* IMBGs genomes resulted in two plasmids (1 and 2) on each strain, with a variation of only 20 nucleotides in one of the contigs of plasmid 2. Although only plasmid 1 was confirmed as circular, plasmid 2, which contains two contigs, was characterized as a mob conjugative plasmid (Table 3).

**TABLE 3:** General features of *K. oboediens* plasmids prediction in samples of space/Mars-like (IMBG185) conditions and reference ground (IMBG180) samples.

Features	IMBG185 (space/Mars-like conditions)			IMBG180 (reference ground)		
Number of plasmids	2			2		
Plasmid name	P1	P2		P1	P2	
Number of contigs	1	2		1	2	
Contig name	>42	>7	>48	>44	>9	>48
Length of contigs	3,080	167,530	1,294	3,080	167,510	1,294
Total length	3,080	168,824		3,080	168,804	
Coverage	151.80x	1.10x	10.77x	142.26x	1.14x	9.66x
Circular form	+	–	–	+	–	–
Relaxase type	–	MOBF		–	MOBF	
MPF type	–	MPF I		–	MPF I	
Predicted mobility	–	Conjugative		–	Conjugative	

*P1, plasmids 1; P2, plasmids 2; +, present; −, absent.*

### IMBG180 and IMBG185 Show Two CRISPR-Cas Cassettes, but the Locations of the *cas* Genes Differ

*Komagataeibacter oboediens* IMBGs strains genome exhibited two CRISPR-Cas cassettes, which were classified as CAS-VI-B and one as CAS-III-D (or Type I, according to Makarova et al., 2020) subtype. The only difference between the isolates was the position of some *cas* genes along the genome sequence (Supplementary Material 4).

### Moderate Difference in Genome-Specific Genes and Single Nucleotide Variations

The comparison of IMBG genomes resulted in a list of 3,234 corresponding genes present in both samples. This represents 98.5% (3,234/3,282) of IMBG185 (space/Mars-like) and 98.7% (3,234/3,275) of IMBG180 (ground reference) coding sequences. The IMBG185 genome exhibited 48 putative genome-specific genes and the IMBG180 genome displayed 41 putative specific genes. Furthermore, among the corresponding genes, only two of these genes presented SNVs: pyruvate phosphate dikinase (*ppdk*) (1 non-synonymous SNV), and sugar phosphatase (*yidA*) (3 synonymous SNVs) genes (Table 4). The plasmid CDS SNV analysis did not find any variation between the strains, indicating that the CDS plasmid sequences of both strains were identical. The non-synonymous substitution SER285 (transition) that was identified in the *ppdk* gene of IMBG185 was further analyzed with protein modeling and molecular dynamics in order to evaluate if this SNV could affect the predicted binding capacity of the ADP molecule and pyruvate metabolism.

**TABLE 4:** Four single nucleotide variations detected in IMBG185 strain as compared to IMBG180.

Gene	Number of SNVs	Genome	Nucleotide change	Mutation type	Codon position	Substitution type	AA change
*ppdK*	1	185	A	Transition	1	Non- synonymous	S
		180	G				G
*yidA A2*	3	185	C	Transition	3	synonymous	–
		180	T				
		185	T	Transversion	3	synonymous	–
		180	A				
		185	G	Transition	3	synonymous	–
		180	A				

*−, absent.*

### SER285 in Pyruvate Phosphate Dikinase May Not Affect ADP Binding and Pyruvate Metabolism

**FIGURE 5:**
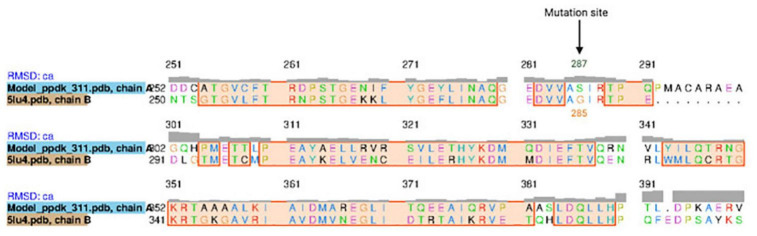
Sequence alignment between *K. oboediens* IMBG185 PPDK and 5LU4 template, emphasizing the ADP/Mg^++^ binding site. The black arrow indicates the mutation site Glycine/Serine.

**FIGURE 6:**
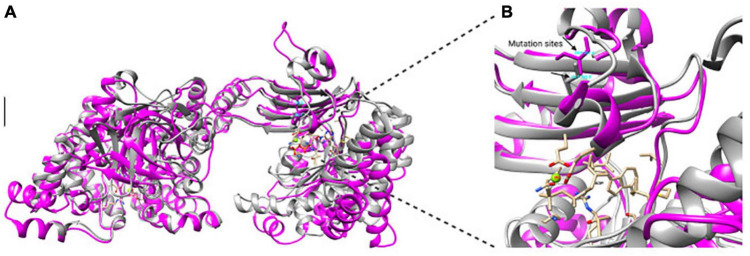
Structural alignment between *K. oboediens* IMBG185 PPDK (magenta) and 5LU4 (dark gray). **(A)** In the overall alignment, both chains displayed similar conformation. **(B)** Detailed ADP/Mg^++^ binding site showing both SER287 (magenta) and GLY285 (dark gray) amino acids in similar positions between the two enzymes, as well as the cavity conformation.

The *K. oboediens* PPDK 3D model was constructed and structurally aligned with its respective template (5LU4). The region where the mutation occurred is well-aligned with its template corresponding amino acids (5LU4 ALA 255 to HIS 390), which are involved with binding to ADP and Mg^++^ ligands (Minges et al., 2017a,b; Figure 5). RMSD between *K. oboediens* PPDK chain B and 5LU4 chain A was 0.93 Å with overall identity of 57%. Figure 6 clearly shows the structural alignment between *K. oboediens* PPDK (magenta) and 5LU4 (dark gray) with the amino acids SER287 and GLY285 (respectively on nearby positions) as they appear surrounding the ADP/MG^++^ binding site. Additionally, the amino acids responsible for binding to these ligands in the ADP/MG^++^ binding site are in the same positions between the two proteins.

**FIGURE 7:**
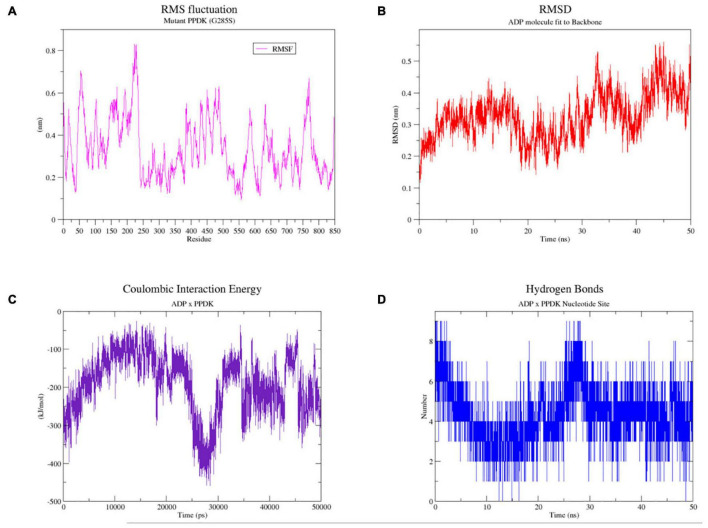
Molecular dynamics results for 50 ns of simulation of the 5LU4 mutated (G285S) in complex with ADP and Mg^++^. **(A)** RMS amino acid fluctuation graph for the 5LU4 chain B during the 50 ns simulation. **(B)** RMSD graph of the ADP ligand bound to the 5LU4 backbone during all the MD simulation. **(C)** Short-range coulombic energy graph of interaction between the ADP ligand and 5LU4 enzyme. **(D)** Hydrogen bond graph for the interaction between the ADP molecule and the 5LU4 amino acid side chains.

After 50 ns of solvent-accessible molecular dynamics of the complex 5LU4 mutated G285S, the behavior of the ADP ligand and the influence of serine on the nucleotide-binding positioning were evaluated. The root mean square fluctuations (RMSF) of all the chain B amino acids were analyzed (Figure 7A), displaying that the amino acid S285 had an RMSF of 0.3 nm, which indicated that its sidechain possibly had freely rotational movements. This observation was further confirmed by the MD video (see Supplementary Material 5^10^), showing the rotational movements of this amino acid. Additionally, the binding stability of the ADP molecule to the protein backbone (Figure 7B) was also evaluated, revealing that the root mean square deviation of this ligand was below 0.4 nm, which indicates that the ligand is stable inside its binding site. Furthermore, the MD video also confirmed the ADP positioning inside its 5LU4 binding site for all the 50 ns time of the simulation. The Coulombic interaction energy between ADP and 5LU4 (Figure 7C) showed that they had a high affinity with each other, with an average of −191.586 KJ/mol. Therefore, it is unlikely that they have been affected by the mutated SER285 amino acid. Furthermore, ADP formed a high number of hydrogen bonds (Figure 7D) with the enzyme active amino acids, varying from 1 to 8, during all the simulation. Altogether, it is unlikely that the mutated SER285 can affect the binding capacity of the ADP molecule or the enzyme metabolism.

### Difference in Total Gene Content Does Not Affect Metabolic Profiles of *Komagataeibacter oboediens* IMBG180 and IMBG185

The genome-scale metabolic reconstructions of *K. oboediens* species comprised 1,894 enzymatic reactions associated with 1,111 genes and 1,418 metabolites (small-molecules in the MetaCyc ontology), covering 33.9% of the annotated open reading frames (Supplementary Material 6). Furthermore, we evaluated differences of the metabolic repertoire between the two isolates of *K. oboediens* IMBG180 and IMBG185 using KEGG and MetaCyc ontologies for metabolic reconstructions.

**FIGURE 8:**
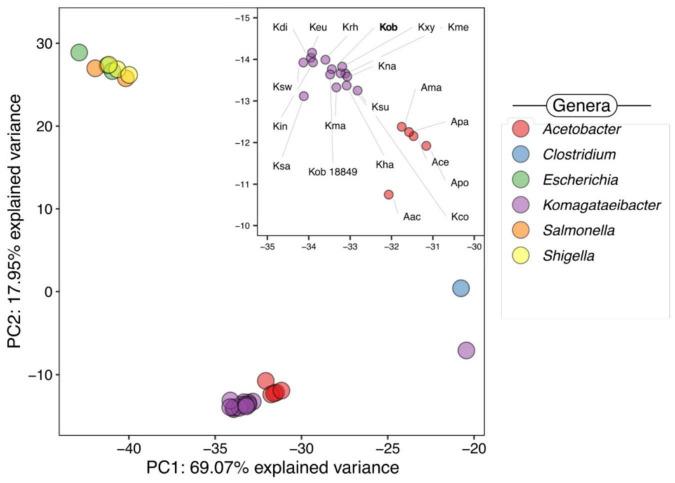
Principal component analysis based on KEGG enzymatic predictions of *Komagataeibacter* genus relating acetic acid bacteria and Enterobacteriaceae representatives. *Acetobacter aceti* (Aac), *A. cerevisae* (Ace), *A. malorum* (Ama), *A. pasteurianus* (Apa), *A. pomorum* (Apo), *Clostridium kluyveri*, *Escherichia coli*, *E. albertii*, *E. fergusonii*, *K. cocoi* (Kco), *K. diospyri* (Kdi), *K. europaeus* (Keu), *K. hansenii* (Kha), *K. intermedius* (Kin), *K. kakiaceti* (Kka), *K. maltaceti* (Kma), *K. medellinensis* (Kme), *K. nataicola* (Kna), *K. oboediens* LMG 18849 (Kob 18849), *K. rhaeticus* (Krh), *K. saccharivorans* (Ksa), *K. sucrofermentais* (Ksu), *K. swingsii* (Ksw), *K. kylinus* (Kxy), *Salmonella bongori*, *S. enterica*, *Shigella boydii*, *Sh. flexneri*, and *Sh. sonnei*.

The genome-based analysis revealed the absence of gene content differences that could impact the metabolic capacities of post-flight *K. oboediens* IMBG185, since the predicted content of metabolic genes of the ground reference (IMBG180) and strains exposed to space/Mars-like conditions (IMBG185) was quite similar, independent of the metabolic ontology applied (KEGG or MetaCyc) (Supplementary Material 6). Subsequently, we compared the metabolic functions within the *Komagataeibacter* genus, as well as how it compares to other bacteria. As expected, there is extensive conservation, in terms of metabolic capacities, within the genus (Figure 8). Furthermore, bacteria with similar metabolic profiles are also closely related to *Komagataeibacter*, particularly the acetic acid bacteria within the *Acetobacter* genus (Figure 8). We also included in these analyses representatives from Enterobacteriaceae, which clustered together in a single group but have strong metabolic dissimilarities when compared to the acetic acid bacteria (Figure 7), probably reflecting different lifestyles of these organisms. It is worth noting that *K. kakiaceti* was the only *Komagataeibacter* representative that did not group with the remaining bacteria of this genus (Figure 8). Next, we evaluated pathways of interest coded in the genomes of *K. oboediens* IMBGs that could be important to the overall functioning of the KMC.

### Small Difference Found in Central Carbohydrate and Energy Pathways

The main pathways found in IMBGs as glycolysis, pyruvate oxidation, Krebs cycle, pentose phosphate pathway (oxidative and non-oxidative phases), phosphoribosyl diphosphate (PRPP – an important intermediate in cellular metabolism), and Entner-Doudoroff pathway had their KOs completely matched (100%) with the reference pathway of KEGG. Six of seven of the gluconeogenesis potential proteins were detected (86%), and only one protein was not complete (86%) considering the pentose phosphate pathway (7 proteins). Other carbohydrate pathways found were the De Ley-Doudoroff pathway (D-galactonate degradation), nucleotide sugar biosynthesis, and UDP-N-acetyl-D-glucosamine biosynthesis (Supplementary Material 7).

### Fermentative Pathways Common to IMBG180 and IMBG185

For both IMBGs, several fermentative complete pathways were identified through the genome potential enzymes. Key enzymes of the fermenting processes were found, such as phosphate acetyltransferase (*pta*) and acetate kinase (*ackA*) for acetate production, pyruvate decarboxylase (*pdc*) and alcohol dehydrogenase (*adh*) for ethanol production, acetolactate synthase (*ilv*) for 2-acetolactate production, α-acetolactate decarboxylase (*budA*) and diacetyl reductase (*budC*) for acetoin production, and finally the enzyme diacetyl reductase (*budC*) to complete the acid homeostasis in cells (Supplementary Material 7).

### No Difference Is Observed in Medium-Chain Carboxylate Pathways

The putative presence of genes related to the fatty acid biosynthesis (FAB) route for MCCA synthesis such as acetyl-CoA carboxylase (*accABCD*) (all the subunits) and ortholog coding for malonyltransferase (*fabD*) (at least one), ketoacyl-ACP synthase (*fabF; fabH*), ketoacyl-ACP reductase (*fabG*), hydroxyacyl-ACP dehydratase (*fadZ*), and enoyl-ACP reductase (*fabI*; *fabK*) enzymes was detected (Supplementary Material 8).

### Stress-Related Pathways Are Intact in Both Genomes

In order to disclose general stress-related genes coded in IMBGs genomes, we firstly conducted a large-scale analysis by leveraging information on all the stress-associated, manually reviewed proteins available in UniProtKB/Swiss-Prot and compared both sequenced strains. This resulted in the identification of highly conserved genes in IMBGs genomes (Supplementary Material 9), and no differences in gene content between both IMBGs were observed in this analysis. Genes with chaperone activities, such as *groL, groS, dnaJ, dnaK*, and *hfq* were detected, as well as the products of *recA* and *uvrABC* systems involved against DNA damage and other resistance properties. Protease activities coded by *hslVU*, *lon*, and *clp* were detected in the genomes, and also *hrcA* and *lexA*, both of which regulate the expression of part of these genes. Trehalose biosynthesis pathway (*otsA/otsB*) genes associated with ROS scavenging (*katB*, *oxyR*) were also identified in both genomes (Supplementary Material 9).

### No Topological Change in Cellulose Biosynthesis Operons

The cellulose synthesis key *bcs* operons following the *K. xylinus* (a close member of *K. oboediens*) as described by Liu et al. (2018), Gullo et al. (2019) were constructed and can be accessed on Gullo et al. (2019), La China et al. (2020). There was no topological change in any of the *bcs* operon genes between postflight and reference genomes, and all the genes of the four operons had 100% identity at both the DNA and amino acid levels, as recently characterized by Lee et al. (2021).

### Equal Number of Nitrogen-Fixing Genes Found in Both IMBG180 and IMBG185

A total of five protein-coding genes associated with biological nitrogen fixation was retrieved in both genomes of IMBG185 (space/Mars-like conditions) and IMBG180 (ground reference) strains (Table 5). One gene (*nifU*) product is involved in the assembly and incorporation of iron into the metalloclusters of a nitrogenase, whereas the other four genes either encode transcription factors that regulate (positively and negatively) nitrogenase (such as *fixK* and *vnfA*) or code for proteins that regulate the transcription factors of nitrogenase (such as the PII proteins *glnB* and *glnK*).

**TABLE 5:** Genes associated with biological nitrogen fixation in ground reference (IMBG180) and space/Mars-like conditions (IMBG185) genomes.

Gene code	Description	Protein function
*nifU*	Nitrogen-fixing thioredoxin-like protein	Synthesis of metalloclusters of nitrogenase
*fixK*	Nitrogen fixation regulation protein	Regulation of the expression of nitrogen fixation
*vnfA* (*rtcR*)	transcriptional regulatory protein	Regulation of the Vanadium-nitrogenase
*glnB*	Nitrogen regulatory protein P-II 1	Regulation of transcription factors of Nitrogen-fixing genes
*glnK*	Nitrogen regulatory protein P-II 2	Regulation of transcription factors of Nitrogen-fixing genes

### No Change Is Observed in Hopanoid Lipid Biosynthesis Gene Number or Gene Sequence

There are five enzyme-coding genes directly associated with the biosynthesis of hopanoid lipids in both genomes (Table 6). One of the genes (*sqc*) codes for the enzyme responsible for the synthesis of hopanoid lipids, whereas another gene (*hpnR*) encodes an enzyme that modifies these hopanoids. Moreover, the other three genes (*hpnC, hpnD*, and *hpnE*) code for enzymes that jointly participate in the conversion of all trans-farnesyl diphosphate to squalene.

**TABLE 6:** Genes associated with biosynthesis of hopanoid lipids ground reference (IMBG180) and space/Mars-like conditions (IMBG185) genomes.

Gene code	Description	Protein function
*sqc*	Squalene-hopene cyclase	Synthesis of diploptene or diplopterol (C30 hopanoids)
*hpnR*	hopanoid C-3 methylase	Methylation of hopanoids at the C3 position
*hpnC*	hydroxysqualene synthase	Conversion of all-trans-farnesyl diphosphate to squalene (three-step reaction)
*hpnE*	hydroxysqualene dehydroxylase	Conversion of all-trans-farnesyl diphosphate to squalene (three-step reaction)
*hpnD*	presqualene diphosphate synthase	Conversion of all-trans-farnesyl diphosphate to squalene (three-step reaction)

## Discussion

A growing body of evidence indicates that exposure of bacteria in culture to the spaceflight environmental stressors results in stress-induced mutations (Li et al., 2014; Guo et al., 2015; Fajardo-Cavazos et al., 2018; Zhao et al., 2019). Direct damage of DNA by cosmic radiation and indirect consequences due to reactive oxygen species generation affect the genetic apparatus of exposed microorganisms. Genomics methods revealed a number of mutant microbial strains after spaceflights. For instance, in Gram-positive bacteria *Bacillus subtilis* (Fajardo-Cavazos et al., 2018), *Staphylococcus aureus* (Guo et al., 2015), and Gram-negative *Klebsiella pneumoniae* (Li et al., 2014), many differences were found between spaceflight and ground control samples. Conversely, comparative genomic analysis of *Acinetobacter baumannii* flight (33 days on the Shenzhou 11 spacecraft) strain yielded only a few mutations compared with the reference strain (Zhao et al., 2019). Only one SNV was identified in the flight strain, suggesting that these strains underwent the genomic changes in short-term spaceflight.

Simulated Martian conditions on the ISS also induced mutagenesis in dehydrated bacterial material. After a 1.5-year exposure to Mars-like conditions, the rates of induced mutations to rifampicin resistance and sporulation deficiency in *B. subtilis* spore DNA increased (Moeller et al., 2012). In the BIOMEX project, KMC samples were exposed to space/Mars-like conditions, and genomes of re-isolated *K. oboediens* from KMC (ground reference strain IMBG180 and space exposed strain IMBG185) were remarkably similar (Table 2 and Supplementary Material 2), indicating that *K. oboediens* tolerated extra-terrestrial conditions.

Genomes of *K. oboediens* strains show some differences in genome size and rRNA + tRNA (*K. oboediens* 174Bp2 and: ∼3.56 Mpb and 96, *K. oboediens* LMG 18849: ∼4.18 Mpb and 51, *K. oboediens* BPZTR0: 2,64 Mbp and 54) (Andrés-Barrao et al., 2011; Ryngajłło et al., 2019; Pinar et al., 2020), and most of these differences in the same species of *K. oboediens* are probably due to transferable mobile elements DNA (Darmon and Leach, 2014). In both samples of IMBGs, we also observed a similar trend and not much difference at genomic level (genome size ∼3.56 Mpb and the rRNA + tRNA is 51–52) (Tables 2, 3).

Using the genomic islands classification proposed by Soares et al. (2016), we have predicted four symbiotic, four metabolic, seven virulence + pathogenicity, and six antibiotic resistance islands in both genomes. We identified symbiosis island genes in both the strains encoding nitrogen fixation proteins such as nitrogen fixation regulation protein (FixK), molybdenum permease, and Fe^3+^ siderophore transport system. *Gluconobacter kombuchae* (now *K. hansenii*) isolated from Kombucha tea has been shown to have nitrogen-fixing capabilities (Dutta and Gachhui, 2007). Nevertheless, putative nitrogen fixation/regulation genes such as *nif*H in *K. hansenii* JR-02 (Li et al., 2019) and *nifU, ntrB, ntrC*, *ntrX*, and *ntrY* in BC producer *K. rhaeticus* ENS9a are reported, which are not homologous to *nif* HDK of *Klebsiella oxytoca* or other nitrogen-fixing bacteria. In *K. oboediens* IMBG180, no structural genes of nitrogenase were found, and, to date, no experimental evidence of N-fixation by these species was reported. Free-living bacteria carry the *nif*-genes originated from symbiotic ancestors (carrying both *nif-* and *nod*-genes) (Tao et al., 2021), and this transition is characterized by losses of symbiosis-related genes, most of which were in the symbiosis island. This loss probably occurred in *K. oboediens*, which now harbors only remnants of *nif*- and *nod*-genes clusters.

Antimicrobial activity through bacteriocins has been detected in lactic acid bacteria isolated from kombucha (Matei et al., 2018; Pei et al., 2020). Thus, the acquisition and maintenance of antibiotic resistance genes probably through phages by KMC may be necessary (Góes-Neto et al., 2021), which could be supported by the presence of antibiotic resistance islands found here.

Genomic islands often harbor transposases that affect the activity of nearby genes (Soares et al., 2016). A thermo-adapted strain of *K. oboediens* MSKU 3 (39°C) isolated from vinegar displayed, among other mutations, a transposon insertion into cellulose synthase catalytic subunit homolog (MSKU 3_1013) (Taweecheep et al., 2019), showing the stimulation of transposase in higher temperature. In both of our strains, the number of transposase genes was 39 (Supplementary Material 4), while 47 were found in *K. oboediens* 174Bp2 (Andrés-Barrao et al., 2011). The reason behind the smaller number of transposons in our samples is unknown.

Prophages can provide both resistance mechanisms as well as some metabolic advantages, allowing the bacteria more chances to survive (Fortier and Sekulovic, 2013). Furthermore, phenotypic diversity among bacterial strains of the same species is typically connected with the mobile genome elements of DNA (Darmon and Leach, 2014). Three intact prophages were found in both IMBGs genomes, which is in accordance with the range retrieved by Ryngajłło et al. (2019) who reported 0–4 intact prophages in three *Komagataeibacter* species from distinct clades of *K. oboediens*. Some differences between IMBG185 and IMBG180 strains for the prophage profile were also found, and interestingly, 18 genes similar to *Streptomyces* phage were uniquely found in IMBG185 (Figure 3 and Supplementary Material 10).

Plasmids appear to have a high mobility within the *Komagataeibacter* genus. In our study, two plasmids (P1 and P2) were predicted in both strains (Table 3) which is similar in number reported in *K. xylinus* DSM 2325 (Jang et al., 2019). Nonetheless, *Komagataeibacter* species show a variable number of plasmids that ranges from 3 to 14 (Ogino et al., 2011; Kubiak et al., 2014; Zhang et al., 2017; Lu et al., 2020; Ryngajłło et al., 2020). Plasmid P1 in our samples is circular; however, relaxase and *mpf* sequences were not found. Therefore, this plasmid is probably non-mobilizable in nature. On the other hand, plasmid P2 exhibits a linear form and has a set of mobility (*mob*) and mating pair formation (*mpf1*) genes that are essential for conjugation and self-transmission (Smillie et al., 2010).

Conversely, (Ryngajłło et al., 2019) solely detected CRISPR/Cas system of the Type I–E (Cas3) in only *K. hansenii* and *K. medellinensis* NBRC; both our *K. oboediens* strains display Cas proteins cassettes of two distinct subtypes: CAS-VI-B and CAS-III-D subtypes (although Makarova et al., 2020 classified the latter as Type I) and located in different positions in the genomes (Supplementary Material 10). To date and to our knowledge, it is the first time the subtype CAS-VI-B has been described for this genus. The CRISPR-Cas system maintains the genome stability in acetic acid bacteria, such as *Acetobacter pasteurianus* (Wang et al., 2015). Therefore, the presence of CRISPR-Cas system in IMBGs suggests that mobile genetic elements are, at least, under control of this specific protective molecular system. Nonetheless, additional studies are required to investigate whether the predicted cassettes are rare subtypes of CRISPR/Cas systems and whether they are functional.

Prions can serve as heritable bet-hedging devices for diversifying microbial phenotypes (Newby and Lindquist, 2013). The bacterial transcription terminator Rho of *Clostridium botulinum* (Cb-Rho) could form a prion and expresses amyloidogenicity (Yuan and Hochschild, 2017). Despite protein aggregates being detected in IMBGs cultures (data not shown), and the transcription terminator Rho gene being detected, the Cb-Rho cPrD domain was not found in the transcription terminator Rho gene of IMBG180 and IMBG185 genomes.

Another interesting finding is the presence of protein-coding genes associated with hopanoids biosynthesis in both the genomes. Hopanoid lipids might have a crucial importance in tolerance of space/Mars-like stress conditions, since KMC UV-irradiated samples displayed the highest read relative abundance of the protein coding gene squalene-hopene cyclase (*shc*) (Góes-Neto et al., 2021). In fact, both *K. oboediens* IMBGs (derived from KMC) not only contain *shc* but also other genes involved in the synthesis and modification of hopanoids. Moreover, as hopanoids can intercalate into lipid bilayers of membranes because of their both planar and hydrophobic structure, they may also decrease cellular permeability to oxygen (Poger and Mark, 2013), which could cause a decrease of the intracellular oxygen concentration.

Hopanoid-enriched membranes may facilitate aerobic nitrogen fixation by providing a physical barrier that restricts oxygen diffusion and may permit the maintenance of low intracellular O_2_ concentrations (Cornejo-Castillo and Zehr, 2019). Furthermore, the typical KMC cellulose biofilm mainly formed by cellulose-producing bacteria (such as *K. oboediens*) may also act not only as a protection of the liquid phase from colonization by competitors but also as a physical barrier to oxygen diffusion to that phase (May et al., 2019; Góes-Neto et al., 2021), creating a microaerophilic environment that would be compatible with nitrogen fixation. Taken together, KMC may be a good candidate system for future Mars colonization.

Finally, in our study, *K. oboediens* did not exhibit considerable genomic changes after exposure to a space/Mars-like environment. One possible explanation could be that the *K. oboediens* genome was not affected by the Mars-like conditions experienced outside the ISS due to biofilm and mineral shielding of latent bacterial cells in KMC. Cellulose-based biofilm is considered a protective shield against environmental cues, including UV (Williams and Cannon, 1989). Despite the stability of genomic apparatus under the exposure experiment, *K. oboediens* exhibited altered fitness after spaceflight that expressed a slower growth rate and cellulose synthesis (Orlovska et al., 2021), a changed rate of enzyme activities (dehydrogenases, nucleases), cellular membrane lipidome structure, and a mode of outer-membrane vesicle production (Podolich et al., 2020). Since genomic content does not show extensive changes in Mars-like conditions, we may assume that the set of changes observed in *K. oboediens* are associated with cytoplasmically inheritable damage but still have functional biomolecules and protein aggregates, which are associated with the impact of environmental stressors (Govers et al., 2018; Wang et al., 2020). Additionally, the lack of mutations in *K. oboediens* from KMC biofilm exposed to space or Mars-like conditions also correlates with conserved core microbiome of KMC exposed to the same conditions as observed in our recent study (Góes-Neto et al., 2021). Although the mutations found do not seem to affect the genomes, as well as the maintenance and conservation of the metabolic pathways, we suggest that the genome is stable under space conditions. Nonetheless, future studies are needed to support this claim.

## Conclusion

Our study suggests that the genome of *K. oboediens* either is stable in KMC exposed to space or that space-exposed KMC recovers most of its functions after upon successive cultures in the lab after return from space. Comprehensive comparative genomics of two strains: *K. oboediens* IMBG180 (ground-based reference) from the laboratory-kept KMC sample and *K. oboediens* IMBG185 (space-exposed) from post-flight KMC revealed that their genomes are highly similar both structurally and functionally, without any striking changes. Both the *K. oboediens* IMBG genomes harbor hopanoid lipids as well as nitrogen-fixing genes, which may make a microaerobic environment compatible with biological nitrogen fixation. The observed genome integrity of *K. oboediens* in the space-exposed KMC samples may be also due to the protecting biofilm of the KMC. Notably, if the biofilm is responsible for protecting the *K. oboediens* genome, this KMC biofilm needs attention to explore its potential for space-related technologies.

## Data Availability Statement

The datasets presented in this study can be found in online repositories. The names of the repository/repositories and accession number(s) can be found in the article/Supplementary Material.

## Author Contributions

NK, DB, and AG-N designed the study. DS, RK, FA, AJ, RP, RD, ST, AC, EA, OK, IO, OP, OR, PR, VD, BB, BA, ATU, DB, and J-PV performed the analyses. DB, DS, ATU, NK, and AG-N wrote, reviewed, and edited the final manuscript. All authors contributed to the article and approved the submitted version.

## Conflict of Interest

The authors declare that the research was conducted in the absence of any commercial or financial relationships that could be construed as a potential conflict of interest.

## Publisher’s Note

All claims expressed in this article are solely those of the authors and do not necessarily represent those of their affiliated organizations, or those of the publisher, the editors and the reviewers. Any product that may be evaluated in this article, or claim that may be made by its manufacturer, is not guaranteed or endorsed by the publisher.

## Footnotes

1
https://www.bioinformatics.babraham.ac.uk/projects/fastqc/

2
https://www.uniprot.org/

3
https://www.ebi.ac.uk/interpro/

4
https://pfam.xfam.org/

5
https://www.ncbi.nlm.nih.gov/

6
https://www.genome.jp/kegg/

7
https://R-project.org

8
https://www.rcsb.org/structure/5LU4

9
https://plasma-gate.weizmann.ac.il/Grace/

10
https://drive.google.com/file/d/1ME25mtaLUbhPYKtFJRKlXGNI_vsAhZN5/view?usp=sharing

## Supplementary Material

Supplementary Material 1 List of shared and specific genes in samples IMBG180 and IMBG185.

Supplementary Material 2 Genome completeness of *K. oboediens* IMBG180 and IMBG185 compared with *K. oboediens* BPZTR01 using a BUSCO analysis.

Supplementary Material 3 Spreadsheet of genomic islands found in both genomes IMBG180 and IMBG185.

Supplementary Material 4 Spreadsheet of prophages with gene annotation.

Supplementary Material 5 Molecular dynamics video of PPDK.

Supplementary Material 6 Annotation, using KEGG ontology, of the KO identifiers for both genomes of *Komagataeibacter oboediens*, IMBG180 and IMBG185.

Supplementary Material 7 Pathway conservation and completeness, evaluated using the KEGG ontology, of strains *Komagataeibacter oboediens* IMBG185 and *K. oboediens* IMBG180. Both genomes share the same set of KO orthologs; for this reason, the key represents conservation across both genomes. Complete pathways are highlighted in bold.

Supplementary Material 8 Orthologs, and respective loci IDs, of key genes related to the synthesis of medium-chain carboxylates (MCAA) through the fatty acid biosynthesis pathway detected in *Komagataeibacter oboediens* genomes IMBG185 and IMBG180.

Supplementary Material 9 Orthologs, and respective loci IDs, of key genes related to the stress response detected in *Komagataeibacter oboediens* genomes IMBG185 and IMBG180.

Supplementary Material 10 Prediction of Cas proteins and classification of CRISPR-Cas system subtypes of ground reference (IMBG180) and space/Mars-like conditions (IMBG185) genomes.
