# Supplementary figures and images for: The Space-Exposed Kombucha Microbial Community Member Komagataeibacter oboediens Showed Only Minor Changes in Its Genome After Reactivation on Earth
Source: Front Microbiol. 2022 Mar 11;13:782175. doi: 10.3389/fmicb.2022.782175 (PMC8970348; doi:10.3389/fmicb.2022.782175)

# BUSCO Assessment Results

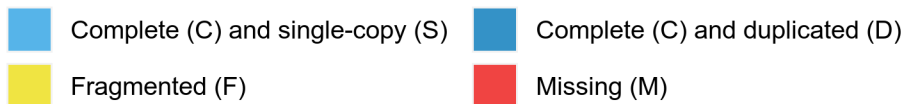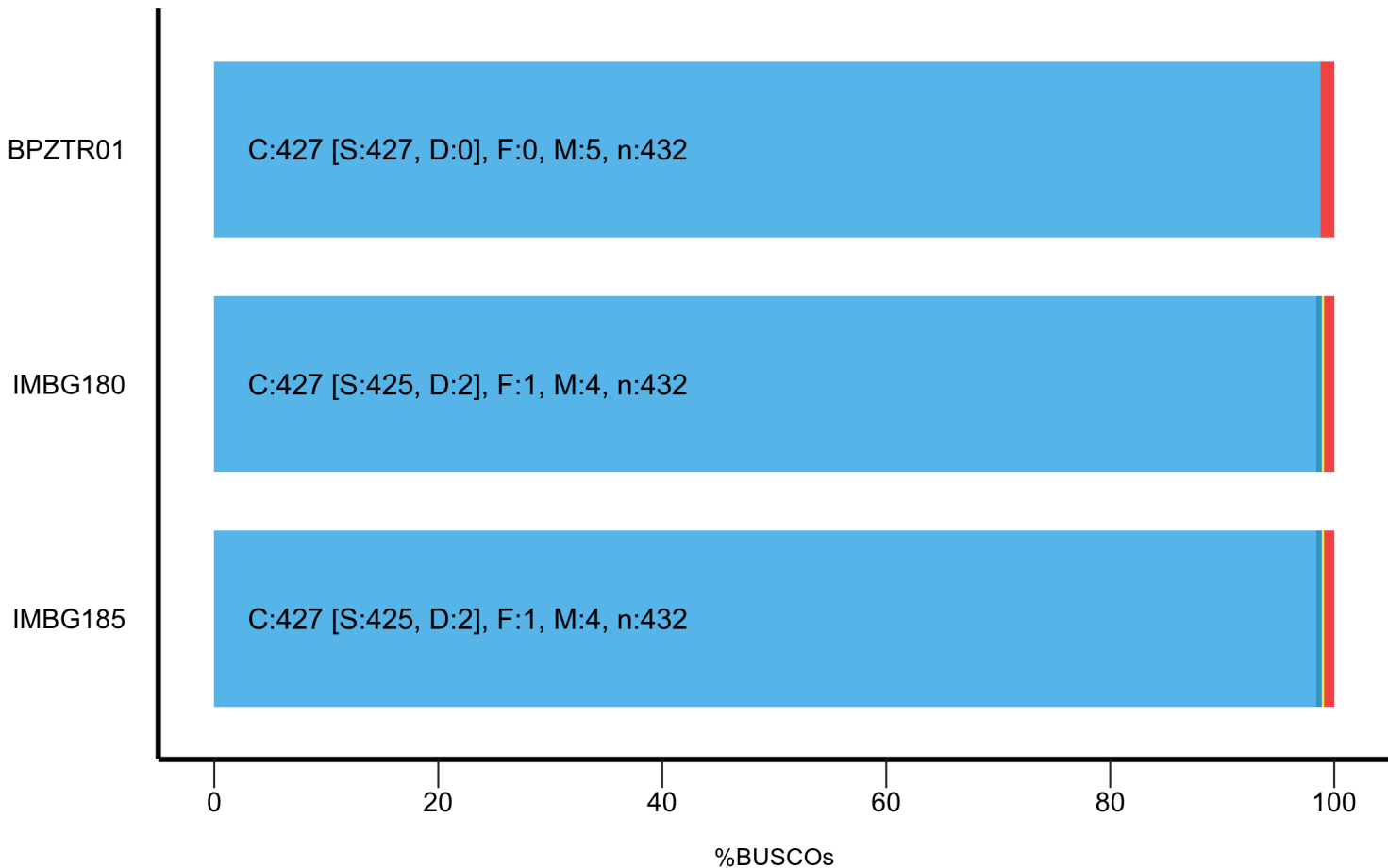

Supplement: Supplementary Material 1 — List of shared and specific genes in samples IMBG180 and IMBG185. [file Data_Sheet_1.PDF]
