# Supplementary material for: The Space-Exposed Kombucha Microbial Community Member Komagataeibacter oboediens Showed Only Minor Changes in Its Genome After Reactivation on Earth
Source: Front Microbiol. 2022 Mar 11;13:782175. doi: 10.3389/fmicb.2022.782175 (PMC8970348; doi:10.3389/fmicb.2022.782175)
Supplement: Supplementary file 9 [file Table_8.DOCX]

| **Strain** | **cassette** | **subtype** | **Cas**  **genes** | **Uniprot ID** | **position** | | **strand** | **bitscore** |
| --- | --- | --- | --- | --- | --- | --- | --- | --- |
|  |  |  |  |  | **start** | **end** |  |  |
| IMBG185 | 1 | CAS-III-D  (or Type 1  Makarova  et al 2020) | *cas3* | G2I4B8 | 11453 | 11801 | -1 | 40.3 |
|  |  |  | *cas3* | G2I4B6 | 11842 | 12057 | 1 | 36.8 |
|  |  |  | *cas7* | F3SF80 | 12160 | 12228 | 1 | 37.7 |
|  | 2 | CAS-VI-B | *cas8* | G2I4T7 | 11706 | 11774 | -1 | 10.0 |
|  |  |  | *csx* | G2I4T5 | 11846 | 11971 | 1 | 10.7 |
|  | 3 | CAS-VI-B | *csx* | F3SD86 | 40667 | 41347 | -1 | 13.2 |
|  |  |  | *cas3* | F3SD89 | 42639 | 45197 | -1 | 19.1 |
| IMBG180 | 1 | CAS-III-D  (or Type 1  Makarova  et al 2020) | *cas7* | F3SF80 | 86422 | 87105 | -1 | 37.7 |
|  |  |  | *cas3* | G2I4B6 | 88135 | 90270 | -1 | 36.9 |
|  |  |  | *cas3* | G2I4B8 | 90695 | 94177 | 1 | 40.3 |
|  | 2 | CAS-VI-B | *csx* | G2I4T5 | 10997 | 11122 | -1 | 10.7 |
|  |  |  | *cas8* | G2I4T7 | 11194 | 11261 | 1 | 10.0 |
|  | 3 | CAS-VI-B | *csx* | F3SD86 | 40667 | 41347 | -1 | 13.2 |
|  |  |  | *cas3* | F3SD89 | 42639 | 45197 | -1 | 19.1 |

Note: Makarova, K. S., Wolf, Y. I., Iranzo, J., Shmakov, S. A., Alkhnbashi, O. S., Brouns, S. J., ... & Koonin, E. V. (2020). Evolutionary classification of CRISPR–Cas systems: a burst of class 2 and derived variants. *Nature Reviews Microbiology*, *18*(2), 67-83.
